# Gut Microbiome of Chinese Forest Musk Deer Examined across Gender and Age

**DOI:** 10.1155/2019/9291216

**Published:** 2019-11-18

**Authors:** Guijun Zhao, Tianyuan Ma, Wenjiao Tang, Diyan Li, Shailendra Kumar Mishra, Zhongxian Xu, Qilin Wang, Hang Jie

**Affiliations:** ^1^Farm Animal Genetic Resources Exploration and Innovation Key Laboratory of Sichuan Province, Sichuan Agricultural University, Chengdu 611130, China; ^2^Chongqing Engineering Technology Research Center for GAP of Genuine Medicinal Materials, Chongqing Institute of Medicinal Plant Cultivation, Chongqing 408435, China

## Abstract

Animal gut microbiota begins to colonize after birth and is functionally indispensable for maintaining the health of the host. It has been reported that gender and age influence the composition of the intestinal microbiome. However, the effects of gender and age on the intestinal microorganism of forest musk deer (FMD) remain unclear. The aim of this study was to establish the relationship between the structure and composition of fecal microbiota of male and female forest musk deer with age. Here, Illumina Miseq 300PE sequencing platform targeting 16S rRNA V3–V4 hypervariable region applied to define the fecal microbiota of male and female FMD with two age groups, juvenile (age 1–2 years) and adult (age 4–10 years). Alpha diversity index did not show significant difference in bacterial diversity between the males and females or among age groups. The intestinal microbiota of FMD was dominated by three phyla, the *Firmicutes*, *Proteobacteria* and *Bacteroidetes* regardless of gender and different ages. Higher proportions of *Proteobacteria* were found in adult male and juvenile female individuals. The composition of *Bacteroidetes* was stable with the gender and age of FMD. Interestingly, the relative abundance of genera *Clostridiales* and *Bacteroidales* were higher in the juvenile FMD. Conversely, proportions of *Pseudomonas* and *Lachnospiraceae* were abundant in the adult FMD. Higher proportions of Ruminococcaceae, *Dore*, and *5-7N15* were found in the juvenile male groups. They may reflect the different immune resistance of male and female individuals at different stages of development. This study explored the fecal microbiota composition of forest musk deer in relation to gender and age, which may provide an effective strategy for developing intestinal microecological preparations and potential musk deer breeding.

## 1. Introduction

The forest musk deer (*Moschus berezovskii*) is one of the six species belonging to Moschidae family which is widely distributed in Asia, except one of the earliest musk deer that have existed from Oligocene deposits in Europe. In China, most forest musk deer is found in Sichuan province [1]. Musk is a substance secreted by the musk gland of adult male individuals during the breeding season to attract females; it not only has high medicinal value, but also is a kind of precious natural superior perfume [2]. Due to habitat loss and heavy poaching the population has drastically reduced, thereby the IUCN listed this species as endangered and protected as a Category I “key” species of wildlife under the national wild animal protection law in China [3]. Since the 1950s, artificial breeding of musk deer has been carried out in China, but the population of breeding musk deer is still difficult to expand. As the forest musk deer is solitary, vigilant, sensitive and timid in the wild, the artificial environment cannot meet their natural needs, resulting in low reproductive capacity, malnutrition and high incidence of intestinal disease in musk deer breading [4].

The gut microbiota begins to colonize in vivo after birth which is essential for maintaining animal health and performance, however, the composition of the intestinal microbiota is thought to change during the aging process [5]. Gut microbes contribute to the host's nutrient absorption and immune response, and can influence the host's behavior [6], development [5], reproduction [7] and overall health [8]. The host genotype, diet, gender, age and geographical environment influence the composition of the intestinal microbiota [9–11]. Recent studies have shown that gender differences in the immune system and the effects of hormonal environments influence the formation of gut microbiota. The interaction between endocrine system and microbiome is helpful to the production of bacteria-assisted hormones and the regulation of host hormone homeostasis [12]. Mueller et al., conducted a meaningful study on intestinal microbiota composition in four European populations in relation to age and gender [13]. They found that healthy males had a higher abundance of *Bacteroides-Prevotella* than females. The vaginal microbial community is dominated by genus *Lactobacillus,* which is known to be regulated by estrogen and colonizes the gastrointestinal tract of girls more often than boys in early life [5]. Microbiota can affect both innate and adaptive immunity, which indirectly reflects the level of disease resistance caused by gender differences [12]. The composition of gut microbiota shifts with different stages of life as well as difference in life style which reflect energy and nutritional needs of the host. Jami et al., (2013) described the effect of age in bovine which has an impact on changes in rumen bacterial communities after birth, indicating that aerobic and facultative anaerobic groups decreased and anaerobic groups increased [14].

Forest musk deer is a ruminant, its unique digestive characteristics and microbial communities help to adapt the food with high fiber content, and play a crucial role in intestinal physiology and regulation. However, the health status of captive musk deer is not good. It has been speculated that inappropriate nutrients and high levels of mineral salts in standard synthetic feeds made them vulnerable to a variety of diseases and disorders [15]. A large number of studies have shown that the imbalance of intestinal microorganisms' homeostasis often leads to the occurrence of intestinal and metabolic diseases [16, 17] . Currently, numerous studies have shown that the diet, health condition and genotype of musk deer have an impact on population of intestinal microorganisms [18–20]. However, the correlation of intestinal microorganisms in forest musk deer with gender and age remain unclear.

Therefore, in this study, we performed high-throughput 16S-rRNA gene sequencing to comprehensively analyze and compare the composition and structure of fecal microbiota in male and female forest musk deer with two age groups. We aimed to identify the differences of the disease resistance between the male and female population of the forest musk deer. The finding of this study will provide a scientific basis for the nutritional microorganism preparation for juvenile and adult forest musk deer, and a theoretical basis for the diagnosis of diseases associated with digestive system to improve the health status and expand the population of the captive forest musk deer.

## 2. Materials and Methods

### 2.1. Sample Collection

There are five juvenile (1–2 years old; JMF1–JMF5), five adult (4–10 years old; AMF1–AMF5) male forest musk deer (MF); and five juvenile (JFF1–JFF5), and five adult (AFF1–AFF5) female forest musk deer (FF) were reared at Chongqing institute of drug cultivation (Sichuan, China). The forest musk deer were fed with constant and balanced diet. Their daily diet consisted of green and fine feed and varied in proportion according to gender and age. The animals included in this study were not administered any antibiotics or other veterinary drugs in the past two months, and each musk deer was kept in its own enclosure. A total of 20 fresh feces sample from FMD were collected during February 2018. To avoid contamination the central portion of feces was placed in sterile polyethylene ziploc bag, wrapped in foil paper, and then quickly dropped into the liquid nitrogen container, finally transferred to −80°C refrigerator, until DNA extraction.

### 2.2. DNA Extraction and 16S rRNA Sequencing

Total genomic DNA was extracted from fecal samples using the TIANamp Stool DNA kit (Tiangen Biotech, Beijing) according to the manufacturer's instruction. The DNA quality was accessed by 1% agarose gel electrophoresis, and DNA concentration was measured by Nano Drop 3300 (Thermo Scientific). The highly variable regions (V3 and V4) of the 16S rRNA gene were amplified using the 338F/806R bar-coded fusion primer set: (338F: 5'-ACTCCTACGGGAGGCAGCA-3', and 806R: 5'-GGACTACHVGGGTWTCTAAT-3'). All PCR was carried out in 25 *μ*l reactions with 5 *μ*l of 5X reaction buffer, 5 *μ*l of 5X GC buffer, 2 *μ*l dNTP (2.5 mM), 1 *μ*l each forward and reverse primer (10 mM), 2 *μ*l gDNA template, ddH2O 8.75 *μ*l, and 0.25 *μ*l Q5 DNA Polymerase. PCR amplification consisted of initial denaturation at 98°C for 2 min, followed by 20–30 cycle of 98°C for 15 s, 55°C for 30 s and 72°C for 30 s, and a final extension of 72°C for 5 min. PCR amplified products were detected by 2% agarose gel electrophoresis and the target fragments were digested and recovered by using AxyPrepDNA gel recovery kit (Axygen). Ultimately, the sequencing Library was prepared by TruSeq Nano DNA library preparation kit from Illumina. The samples were sequenced on Illumina Miseq 300PE sequencing platform at Novogene Bioinformatics Institute.

### 2.3. Bioinformatics Analyses

The raw data of sequencing was stored in FASTQ format. Sequence assembly and quality filtering were performed using FLASH (v1.2.7 : http://ccb.jhu.edu/software/FLASH/) and QIIME (Quantitative Insights Into Microbial Ecology, v1.8.0, http://qiime.org/), respectively to acquire high-quality data. The potential chimeric sequences were removed using USEARCH algorithm (v5.2.236, http://www.drive5.com/usearch/). Then sequences were assigned to operational taxonomic units (OTUs) with a 97% threshold of pairwise identity, and species annotated based on Ribosomal Database Project (RDP)-classifier using the Greengenes reference Database (release 13.8, http://greengenes.secondgenome.com/).

The rarefaction curves and rank abundance curves were displayed with R software, Venn diagrams were created using online weblink (http://jvenn.toulouse.inra.fr/app/index.html). The bar iagram of alpha diversity indices (Chao1 and Shannon) and relative abundance (Phylum and genus) were drawn using GraphPad Prism7. In addition, we used the independent sample *t*-test to analyze the significant difference in diversity index and relative abundance using IBM SPSS Statistics version 19. The Heatmap was generated through R package. The non-metric multi-dimensional scaling (NMDS) was plotted based on the Unweighted Unifrac distance. ANOSIM analysis was conducted for the Unweighted Unifrac distance subject to gender and age. Linear discriminant analysis coupled with effect size (LEfse) was generated to identify the microbial communities differentially represented between the groups at genus level using LEfse software (LDA >2).

## 3. Results

### 3.1. Sequencing Data Quality

**Figure 1:**
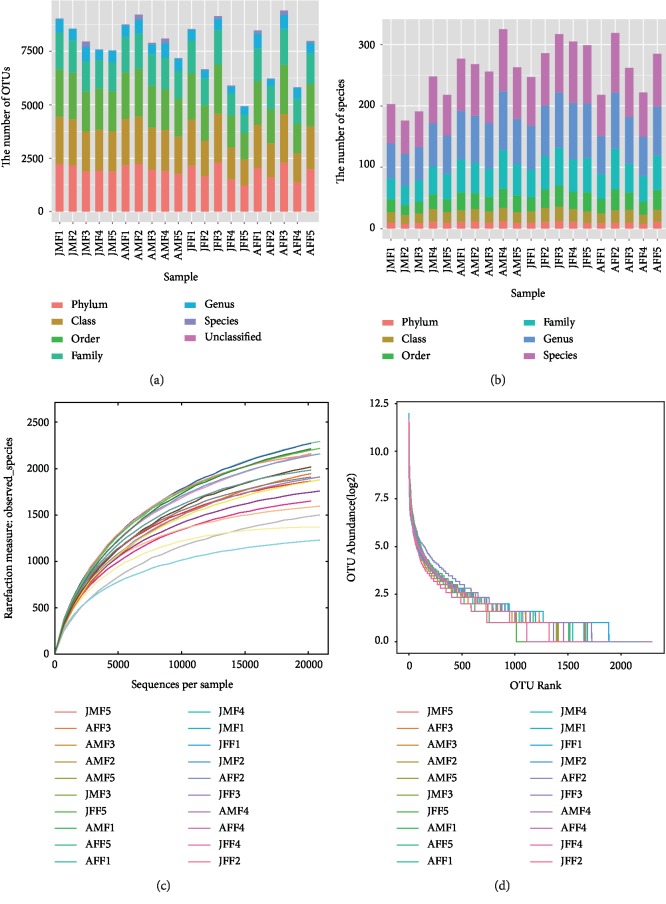
Overview of sequential changes in fecal microbiota composition (a, b). a and b mean that each sample has a different number on a different classification, (c) rarefaction curves, (d) rank abundance curves. The rarefaction curves reflecting the rationality of sequencing depth and diversity of species in feces samples indirectly. In rank abundance curves, wider span of curves reveals richness of species in horizontal direction and the degree of curves showing the evenness of bacterial species in samples vertically.

The Illumina MiSeq 16 s rRNA sequencing data of 20 samples (five from each FMD group of this study) were analyzed for gut microbiota. After performing a series of quality filter steps, a total of 1,076,941 (average length of 422 ± 40 bp) chimera-free high quality sequences were recovered, with an average of 54,392 ± 8145 sequences per sample, ranging from 37,057 to 71,233. These sequences were assigned to a total of 8310 OTUs based on 97% similarity sorted from 20 fecal samples. Each sample has 1906 ± 315 OTUs on average, ranged from 1231–2292 (Figure 1(a)). The sequences were assigned to 16 phyla, 30 classes, 50 orders, 96 families, and 174 genera (Figure 1(b)). The OTUs and species (classified at taxonomic level) for each sample with six classification level are shown in Supplementary [Supplementary-material supplementary-material-1]. The rarefaction curves (Figure 1(c)) became gradually placid with more data indicating that a sufficient number of OTUs were analyzed for each fecal sample to reflect maximum level of bacterial diversity. The rank abundance curves reflecting the richness and evenness of species in fecal samples horizontally and vertically are shown in Figure 1(d).

### 3.2. Diversity, Richness of the Shared Bacterial Communities across FMD Groups

**Figure 2:**
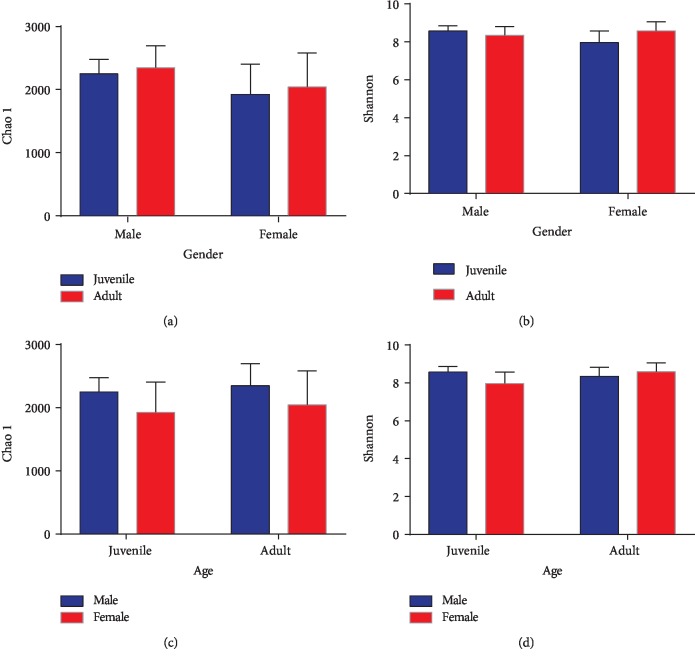
Gender and age-related differences in alpha-diversity index analysis (Chao1 and Shannon). (a, b) The bar graph represents the comparison of Chao1 and Shannon index between juvenile and adult. (c, d) Comparison of Chao1 and Shannon index between male and female forest musk deer.

We compared alpha diversity metrics across four sampling groups of musk deer (JMF-AMF, JFF-AFF, JMF-JFF, and AMF-AFF) to explore the changes in intestinal microbiota. Each musk deer group was divided according to gender and age. The Chao1 and Shannon index were not significant between males, females, or among age groups. However, the patterns of proportional differences for Chao1 diversity index among the groups were recorded as, JMF < AMF, JFF < AFF, JMF > JFF, and AMF > AFF, likewise for Shannon index as, JMF > AMF, JFF < AFF, JMF > JFF, and AMF < AFF (Figure 2). Furthermore, the ACE and Simpson reciprocal diversity index were calculated (Supplementary Tables [Supplementary-material supplementary-material-1]–[Supplementary-material supplementary-material-1]).

**Figure 3:**
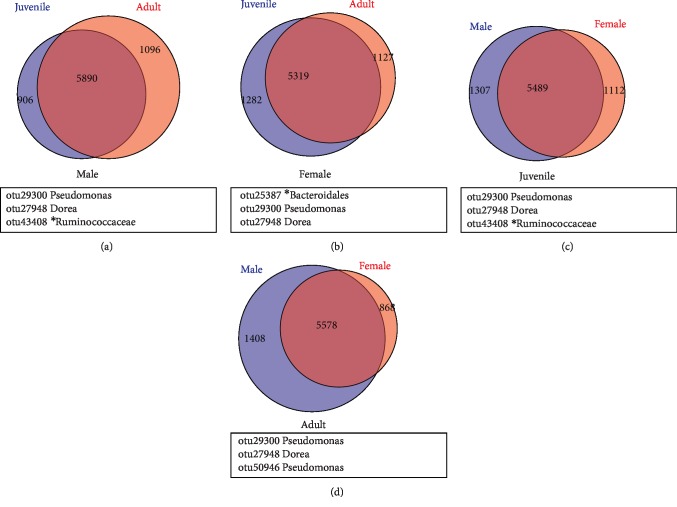
Distribution of bacterial taxa within the four sample groups in Venn diagrams. The numbers in the Venn diagrams represent the unique or common OTUs between the individuals of (a) JMF –AMF (Male), (b) JFF–AFF (Female), (c) JMF–JFF (Juvenile), and (d) AMF–AFF (Adult). The black box below the Venn diagram represents the shared core microbiome.

The Simpson index of JFF was found significantly lower than AFF group (*p* = 0.04). The number of OTUs shared by gender group JMF-AMF and JFF-AFF were 5890, 5319, respectively. Likewise for age group JMF-JFF and AMF-AFF shared OTUs were 5489, 5578, respectively (Figures 3(a)-3(d)). The core bacterial community structure the intestinal microbiota displayed slightly different between JFF-AFF and JMF-AMF group, mainly because unclassified *Bacteroidales* were found in JFF-AFF instead of unclassified Ruminococcaceae in JMF-AMF group. The JMF-JFF group of FMD possessed similar community structure to AMF-AFF, except for the unclassified Ruminococcaceae, which were only identified in JMF-JFF group (Figures 3(a)-3(d)). The microbial communities of forest musk deer were mostly dominated by *Firmicutes* (*Lachnospiraceae*, Ruminococcaceae), *Proteobacteria* (Enterobacteriaceae) and *Bacteroidetes* (*Bacteroidales*), as shown in Supplementary [Supplementary-material supplementary-material-1].

### 3.3. Age-Related Differences in Bacterial Communities among MF and FF Groups

**Figure 4:**
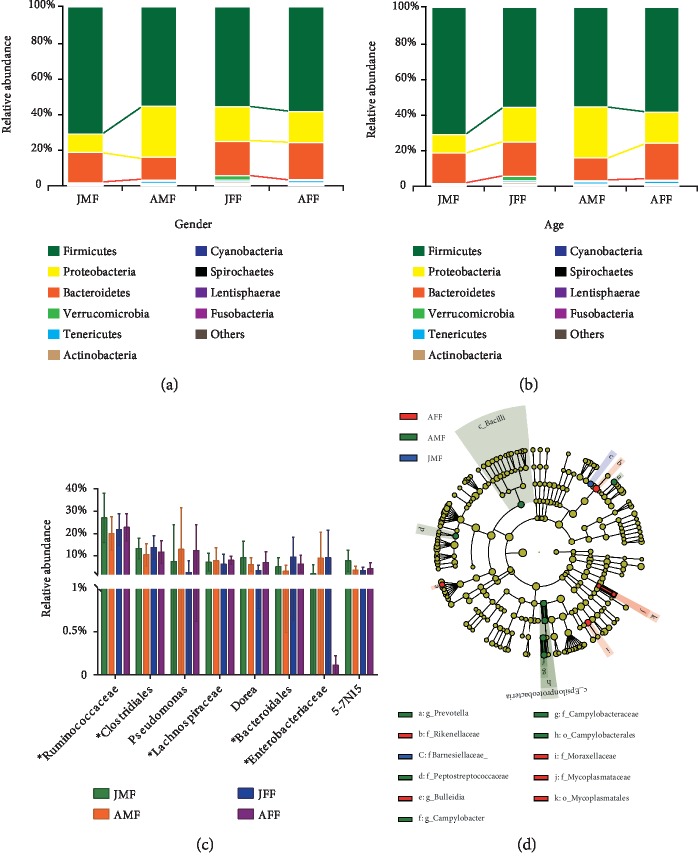
A general overview of microbial composition of different sample groups. (a, b) Phylum level, microbial composition of different groups. (c) Differences in relative abundance of top eight genera (contain the unclassified bacteria indicated with “∗”) among four sampling groups. The significances were determined using the independent-sample *t*-test. (d) Cladogram showing the differences in relative abundance of taxa at five levels between AFF, AMF and JMF. The plot was generated using the online LEfSe software. Red and green circles representing differences in relative abundance between AFF, AMF and JMF and yellow circles mean nonsignificant differences.

Although, there were no significant differences observed in bacterial communities with age, but considerably differed at phylum and genus level. At the phylum level, the *Firmicutes* was most abundant in JMF (71.15%) followed by AFF (58.66%), JFF (55.90%), and AMF (55.66%). On the contrary, the *Proteobacteria* was abundant in AMF (28.67%) followed by JFF (19.43%), AFF (17.45), and JMF (10.35%). The enrichment of *Bacteroidetes* was observed in AFF (20.65%), JFF (19.17%), JMF (17.02%), and AMF (12.59%), as shown in (Figures 4(a) and 4(b), Supplementary Tables [Supplementary-material supplementary-material-1] and [Supplementary-material supplementary-material-1]). At the genus level, Ruminococcaceae was the most predominant family in all four musk deer groups examined in this study. *Clostridiales* was second most prevalent order in juvenile male and female FMD. Amongst all groups, presence of *Pseudomonas* was recorded higher in AMF (12.90%) and AFF (12.31%) in average. The others such as *Lachnospiraceae*, *Dorea*, *Bacteroidales*, Enterobacteriaceae and *5-7N15* were classified with relatively low abundance. Furthermore, Enterobacteriaceae had the lowest abundance in AFF accounting for only 0.11% in average (Figure 4(c), Supplementary Tables [Supplementary-material supplementary-material-1] and [Supplementary-material supplementary-material-1]).

**Figure 5:**
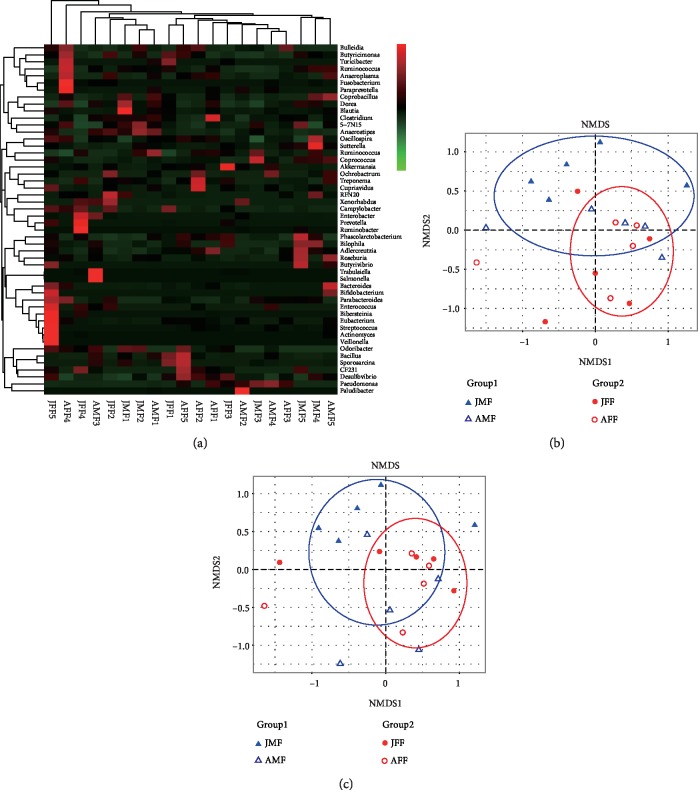
Heatmap and non-metric multidimensional scaling (NMDS) analyses differ in distance between groups. (a) Heatmap analysis results for top 50 genera among the 20 samples based on hierarchical clustering (Unweighted Unifrac distance). Red represents the genus with higher abundance in the corresponding sample, and green represents the genus with lower abundance. (b, c) NMDS plot, the distance was calculated between the samples based on dissimilarity in OTU composition using the Unweighted Unifrac dissimilarity index. Each point represents a sample and a closer distance between two points infers a higher similarity between them. Moreover, the points of different colors belong to different groups.

A LEfSe analysis was performed with the pooled data and observed abundant microbiota composition across 4 musk deer groups with gender and age which might be used as biomarker. Cladogram results showed that a total of 13 genera were differentially represented between JMF, AMF and AFF with LDA scores >2 (Figure 4(d)). Among them, seven bacterial taxa were significantly abundant in fecal microbiota of AMF (e.g., *Bacilli*,* Prevotella*, *Peptostreptococcaceae*, *Campylobacter*, *Campylobacteraceae*, *Campylobacterales* and *Epsilonproteobacteria*) and five genera were significantly abundant in AFF (e.g., *Rikenellaceae*,* Bulleidia*, *Moraxellaceae*, *Mycoplasmataceae*, and *Mycoplasmatales*). Meanwhile, only *Barnesiellaceae* genus was significantly associated to the JMF group. However, we did not find any significantly abundant bacterial taxa in the JFF group. Heatmap results based on Unweighted Unifrac distances for bacterial abundance at genus level showed that each sample varied with the same bacterial genera, but no strong clustering was observed in the samples grouped by gender and age (Figure 5(a)). NMDS did not show strong clustering of samples by gender and age groups, but also showed the existence of certain differences (Figures 5(b) and Figures 5(c)). Furthermore, the analysis of similarity (ANOSIM) revealed that the relative abundance of FMD fecal microbial communities differed among gender groups albeit no differences were found in age groups (*r*2 = 0.0113, *p* = 0.327), which were supported by the NMDS ranking.

## 4. Discussion

This is the first comparative study to characterize the composition and structure of fecal microbiota of forest musk deer with gender and age. Differences in complex fecal microbial communities in male and female is assumed to play important role in intestinal disease development of the forest musk deer. Previously many reports have shown the changes in gut microbiota composition of ruminants, such as Bactrian camel [21], sheep [22], dairy cattle [23]. The microbial composition among various segments of gastrointestinal tract in Bactrian camel accounted for a greater proportion of *Akkermansia* and *Ruminococcaceae* in the large intestine and fecal samples, while *Clostridiales* and *Bacteroidales* were relatively abundant in the forestomach and small intestine [24]. An investigation of the distribution of intestinal flora in small-tail Han sheep showed that *Bacteroidetes*, *Ruminococcus*, *Lactobacillus*, *Flavonifractor* and *Clostridium* were dominant genera in the cecum and rectum, while *Lactobacillus* show a decreasing decreasing trend from jejunum to the cecum [25]. *Firmicutes*, *Bacteroidetes* and *Proteobacteria* predominate in dairy cattle reveal significant spatial heterogeneity in composition, diversity and species abundance distributions of intestinal microbiota [26]. Although, fecal samples do not reflect the dynamics of bacteria throughout the gut, it still reflects the composition of the entire intestinal microbial community [27]. In addition, it is also non-invasive and therefore beneficial for endangered or cryptic species [19]. It is more meaningful for us to find some specific bacteria associated with gender and age groups.

In the four sampling groups of musk deer, we did not find a significant difference in bacterial diversity. However, the abundance and diversity of intestinal microbial community in AFF was higher than JFF, while the diversity in the JMF was higher than AMF in age groups. In gender groups, the abundance and diversity of intestinal microbes in JMF was higher than JFF, while the diversity in AFF was higher than AMF (Figure 2). Previous studies have demonstrated that the gut microbes of Peking ducks become more diverse when they get older [28], and the intestinal microbial composition of piglets is more stable and diversified with increasing age [29]. The mature intestinal environment showed a great diversity of microbial species, however, it was more restricted niche with a more homogeneous bacterial community [29]. A gender specific intestine microbiota study in human reported that the alpha diversity of the underweight group was higher than that of body mass index (BMI) female groups; however, there was no significant difference in alpha diversity between the male BMI groups [30]. These results suggested that microbiota composition can be affected by gender with different BMI level. We inferred from this study that both gender and age can influence the diversity of gut microbes, which is clearly reflected by the difference between the female age group and the juvenile's gender group. The study of alpine musk deer and forest musk deer show that the age difference has little impact on the intestinal microbial diversity, and the inter-species difference is greater than the intra-species difference [19]. Furthermore, the diversity of gut bacteria also increased significantly during the transition from carnivorous to herbivorous. Therefore, genetic background and diet have a greater impact on the diversity of intestinal microorganisms [31].

At the phylum level, the core bacterial phyla belong to *Firmicutes*, *Proteobacteria* and *Bacteroidetes*, which is consistent with previous observations in FMD and other ruminants. The only difference is that the proportion of *Proteobacteria* in this study is higher, but similar to Rex rabbits. In the entire gastrointestinal tract of Rex rabbits, the most dominant phylum was *Firmicutes* followed by the *Proteobacteria* in the foregut [32]. *Firmicutes* accounted for more than 50% in all four sampling groups and up to 71.15% in the JMF group (Supplementary Tables S4 and S5). The ruminants' microflora such as lineages Ruminococcaceae, *Clostridiales*, *Lachnospiraceae* and *Dorea* (Supplementary Tables S6 and S7) were mainly existed in the intestinal bacteria, the *Firmicutes* of forest musk deer. *Firmicutes* were typically the most abundant bacterial phylum in the gastrointestinal tract of vertebrate, particularly in herbivore, which play an important role in the breakdown of the fiber cellulose and nutrient absorption. Most symbiotic bacteria have been reported for the maintenance of intestinal homeostasis and immunity [33]. Meanwhile, the reduction of *Firmicutes* abundance in the intestinal tract of musk deer with diarrhea would likely accompanied by reducing the digestive physiological functions [18]. Interestingly, the *Proteobacteria* was another dominant phylum, but the colonization of this phylum may be variable in different individuals of four sampling groups. The *Proteobacteria* was identified as dominant species in giant pandas and red pandas, which played a key role in digestion of the main food sources of lignin. *Proteobacteria* are often found in the natural environment and individual wild animals may do accumulate more [31, 34, 35]. An increased prevalence of the bacterial phylum *Proteobacteria* is a marker for an unstable microbial community (dysbiosis) and a potential diagnostic criterion for disease. Acute or chronic inflammation and Low fiber diets can lead to enrichment of *Proteobacteria* in the GI tract [36]. In addition, previous study on intestinal microorganisms of diarrhea affected and healthy FMD showed that the diarrhea group had the most bacteria belonging to the Enterobacteriaceae family and was prone to intestinal diseases [18]. The AMF and JFF groups account for a higher proportion of *Proteobacteria* and individual differences. Therefore, we speculated that these two groups may have chronic enteritis, while AFF contains a relatively low level of Enterobacteriaceae, and the group was in a relatively healthy state.*Bacteroidetes* are strictly anaerobic and have the ability to degrade complex molecules (polysaccharides, proteins) in the intestine, which can promote the development of gastrointestinal immune system to improve the nutritional utilization of the host. Therefore, a high-starch diet is beneficial to the enrichment of more *bacteroides*, making them important for both herbivorous and carnivorous diets [37, 38]. Furthermore, we found the composition of *Bacteroidetes* is steady in the individuals of FMD and plays an important role in intestinal digestion.

We also found a competitive relationship between *Firmicutes* and *Proteobacteria*. This relationship was reflected in AMF, which contains more *Proteobacteria* than JMF; JFF contains relatively higher proportion of *Proteobacteria* than JMF, and the AMF contains more *Proteobacteria* than AFF. For the initial three days, the *Proteobacteria* was dominated later on *Firmicutes* begin to rise into the main class in Peking ducks [28]. A prior study found that the ratio of *Firmicutes* to Bacteroides in feces of older pigs (2-, 3-, 6- month) were extremely higher compared to those from piglets at one month of age [29]. In addition, previous studies have shown that the extent of *Proteobacteria* in healthy captive male FMD was 5.2% ± 5.6% and significantly differed among other individuals [39]. Adult males and juvenile females contained more *Proteobacteria*, reflecting similar differences in competitive relationship. Verrucomicrobia mainly had a higher proportion in JFF and lower in other groups and Tenericutes represented mainly in adults. An interesting finding of our study is that the *Clostridiales* and *Bacteroidales* were higher in juvenile than adult FMD. Conversely, *Pseudomonas* and *Lachnospiraceae* were higher in adult. Ruminococcaceae, *Dore* and *5-7N15* were higher in the JMF group than other groups. Members of *Ruminococcus* have been identified as important members of the gut communities in female subjects [40] and this genus is also found more often in female mice [40]. A LEfSe analysis indicated that significant bacteria are present in both age and sex groups. The number of bacteria in the adult group (AMF and AFF) was significantly higher than the juvenile group (JMF and JFF), while male groups (JMF and AMF) found more bacteria with significant differences than female groups (JFF and AFF).

## 5. Conclusion

Overall, this is the first study to demonstrate the impact of gender and age on the gut microbiota of forest musk deer to some extent. This work shows that *Firmicutes*, *Proteobacteria* and *Bacteroidetes* were predominant phyla in the microbial community. There was a competitive relationship between *Firmicutes* and *Proteobacteria* in four sampling groups. The two groups of AMF, and JFF, may be in a sub-healthy state in terms of the diversity and composition of intestinal bacteria with lower diversity and more *Proteobacteria*. This reflects the variable immune resistance of males and females at different growth stages. However, the proportion of *Proteobacteria* in our study was 2 to 4 times higher than previous studies. The present study may provide meaningful biological insights into the age and gender based alterations in fecal microbiota of forest musk deer, and will be useful in developing intestinal microecological preparations and potential musk deer breeding.

## Data Availability

Microbiome data are available at NCBI with the accession number PRJNA541776.

## Supplementary Materials

Supplementary Materials Table S1: Sequencing quality of OTUs and species in each sample; Table S2: The differences in diversity indices (mean ± SD) between Juvenile and Adult forest musk deer; Table S3: The differences in diversity indices (mean ± SD) between male and female forest musk deer; Table S4: The differences in relative abundance (mean ± SD) of major bacterial phyla between Juvenile and Adult forest musk deer; Table S5: The differences in relative abundance (mean ±  SD) of major bacterial phyla between male and female forest musk deer; Table S6: The differences in relative abundance (mean ±  SD) of major bacterial genera between Juvenile and Adult forest musk deer; Table S7: The differences in relative abundance (mean ±  SD) of major bacterial genera between male and female forest musk deer. Figure S1: Classification tree by GraPhlAn.
