# Supplementary material for: Gut Microbiome of Chinese Forest Musk Deer Examined across Gender and Age
Source: Biomed Res Int. 2019 Nov 18;2019:9291216. doi: 10.1155/2019/9291216 (PMC6925676; doi:10.1155/2019/9291216)
Supplement: Supplementary Materials — Table S1: Sequencing quality of OTUs and species in each sample; Table S2: The differences in diversity indices (mean ± SD) between Juvenile and Adult forest musk deer; Table S3: The differences in diversity indices (mean ± SD) between male and female forest musk deer; Table S4: The differences in relative abundance (mean ± SD) of major bacterial phyla between Juvenile and Adult forest musk deer; Table S5: The differences in relative abundance (mean ± SD) of major bacterial phyla between male and female forest musk deer; Table S6: The differences in relative abundance (mean ± SD) of major bacterial genera between Juvenile and Adult forest musk deer; Table S7: The differences in relative abundance (mean ± SD) of major bacterial genera between male and female forest musk deer. Figure S1: Classification tree by GraPhlAn. [file 9291216.f1.doc]

Table S1 Sequencing quality of OTUs and species in each sample

|  |  |  | OTUs |  |  |  |  |  |  | species |  |  |  |  |  |
| --- | --- | --- | --- | --- | --- | --- | --- | --- | --- | --- | --- | --- | --- | --- | --- |
| Sample ID | Paper ID | sequences | Phylum | Class | Order | Family | Genus | Species | Unclassified | Phylum | Class | Order | Family | Genus | Species |
| 4232 | JMF1 | 53224 | 2218 | 2218 | 2218 | 1724 | 629 | 22 | 0 | 10 | 17 | 21 | 34 | 57 | 64 |
| 4236 | JMF2 | 57601 | 2161 | 2161 | 2161 | 1530 | 534 | 4 | 1 | 8 | 14 | 16 | 32 | 52 | 54 |
| 4209 | JMF3 | 60162 | 1877 | 1877 | 1874 | 1411 | 667 | 245 | 2 | 9 | 16 | 19 | 35 | 54 | 58 |
| 4227 | JMF4 | 71233 | 1919 | 1919 | 1919 | 1336 | 474 | 12 | 0 | 11 | 21 | 24 | 45 | 71 | 76 |
| 4196 | JMF5 | 64820 | 1876 | 1876 | 1876 | 1352 | 544 | 6 | 0 | 10 | 17 | 21 | 41 | 63 | 66 |
| 4219 | AMF1 | 42369 | 2174 | 2174 | 2172 | 1646 | 547 | 40 | 0 | 11 | 19 | 29 | 54 | 79 | 85 |
| 4205 | AMF2 | 63307 | 2225 | 2225 | 2223 | 1666 | 641 | 227 | 0 | 12 | 20 | 26 | 49 | 77 | 84 |
| 4199 | AMF3 | 49733 | 1963 | 1963 | 1963 | 1473 | 464 | 68 | 0 | 10 | 18 | 24 | 46 | 74 | 84 |
| 4245 | AMF4 | 54081 | 1911 | 1911 | 1905 | 1476 | 671 | 216 | 1 | 12 | 22 | 31 | 63 | 96 | 101 |
| 4207 | AMF5 | 58507 | 1761 | 1761 | 1760 | 1310 | 558 | 24 | 1 | 9 | 18 | 27 | 51 | 74 | 84 |
| 4233 | JFF1 | 48698 | 2155 | 2155 | 2151 | 1543 | 508 | 21 | 1 | 10 | 18 | 23 | 45 | 72 | 79 |
| 5015 | JFF2 | 57129 | 1660 | 1660 | 1658 | 1248 | 398 | 30 | 1 | 11 | 23 | 30 | 54 | 83 | 85 |
| 4244 | JFF3 | 55837 | 2292 | 2292 | 2288 | 1632 | 518 | 115 | 2 | 12 | 24 | 34 | 62 | 90 | 95 |
| 5010 | JFF4 | 52663 | 1503 | 1503 | 1501 | 1034 | 314 | 39 | 1 | 11 | 21 | 28 | 53 | 92 | 100 |
| 4211 | JFF5 | 55000 | 1231 | 1231 | 1229 | 859 | 365 | 13 | 0 | 9 | 19 | 31 | 56 | 89 | 95 |
| 4224 | AFF1 | 58657 | 2034 | 2034 | 2033 | 1528 | 703 | 140 | 0 | 9 | 16 | 24 | 40 | 62 | 67 |
| 4242 | AFF2 | 44318 | 1598 | 1598 | 1596 | 1083 | 335 | 13 | 2 | 10 | 20 | 34 | 66 | 92 | 97 |
| 4197 | AFF3 | 49060 | 2288 | 2288 | 2287 | 1676 | 660 | 201 | 1 | 10 | 21 | 28 | 47 | 76 | 80 |
| 5001 | AFF4 | 37057 | 1370 | 1370 | 1370 | 1179 | 503 | 24 | 0 | 8 | 15 | 21 | 42 | 64 | 72 |
| 4220 | AFF5 | 43485 | 1991 | 1991 | 1988 | 1428 | 493 | 84 | 1 | 11 | 20 | 32 | 56 | 80 | 86 |

Table S2 The differences in diversity indices (mean ± SD) between Juvenile and Adult forest musk deer

| diversity indices | MF | |  | | FF | |  | |
| --- | --- | --- | --- | --- | --- | --- | --- | --- |
|  | Juvenile | Adult |  |  | Juvenile | Adult |  |  |
| Chao1 | 2248.80±227.47 | 2345.52±348.89 | t=-0.52 | p=0.62 | 1924.41±480.54 | 2044.32±537.44 | t=-0.37 | p=0.72 |
| ACE | 2320.99±216.22 | 2415.04±307.63 | t=-0.56 | p=0.59 | 2022.00±514.81 | 2103.93±561.96 | t=-0.24 | p=0.82 |
| Shannon | 8.58±0.29 | 8.35±0.48 | t=0.93 | p=0.38 | 7.97±0.60 | 8.59±0.47 | t=1.81 | p=0.11 |
| Simpson | 0.98±0.01 | 0.98±0.01 | t=0.75 | p=0.47 | 0.97±0.02 | 0.99±0.01 | t=-2.40 | p=0.04 |

Table S3 The differences in diversity indices (mean ± SD) between male and female forest musk deer

| diversity indices | Juvenile | |  | | Adult | |  | |
| --- | --- | --- | --- | --- | --- | --- | --- | --- |
|  | MF | FF |  |  | MF | FF |  |  |
| Chao1 | 2248.80±227.47 | 1924.41±480.54 | t=1.36 | p=0.21 | 2345.52±348.89 | 2044.32±537.44 | t=1.05 | p=0.32 |
| ACE | 2320.99±216.22 | 2022.00±514.81 | t=1.20 | p=0.27 | 2415.04±307.63 | 2103.93±561.96 | t=1.09 | p=0.31 |
| Shannon | 8.58±0.29 | 7.97±0.60 | t=2.04 | p=0.08 | 8.35±0.48 | 8.59±0.47 | t=-0.80 | p=0.45 |
| Simpson | 0.98±0.01 | 0.97±0.02 | t=2.1 | p=0.07 | 0.98±0.01 | 0.99±0.01 | t=-1.10 | p=0.31 |

Table S4 The differences in relative abundance (mean ± SD) of major bacterial phyla between Juvenile and Adult forest musk deer

| phylum level | MF | |  | | FF | |  | |
| --- | --- | --- | --- | --- | --- | --- | --- | --- |
|  | Juvenile | Adult |  |  | Juvenile | Adult |  |  |
| Firmicute | 71.15%±16.00% | 55.66%±20.53% | t=1.33 | p=0.22 | 55.90%±16.24% | 58.66%±9.36% | t=-0.33 | p=0.75 |
| Bacteroidetes | 17.02%±8.71% | 12.59%±4.07% | t=1.03 | p=0.35 | 19.17%±6.34% | 20.65%±7.31% | t=-0.34 | p=0.74 |
| Proteobacteria | 10.35%±16.63% | 28.67%±23.97% | t=-1.40 | p=0.20 | 19.43%±20.08% | 17.45%±13.37% | t=1.83 | p=0.86 |
| Verrucomicrobia | 0.17%±0.17% | 0.78%±1.02% | t=-1.33 | p=0.25 | 2.57%±3.91% | 0.61%±0.56% | t=1.11 | p=0.33 |
| Tenericutes | 0.58%±0.30% | 1.17%±0.76% | t=-1.60 | p=0.17 | 0.90%±0.56% | 1.01%±1.06% | t=-0.21 | p=0.84 |
| Actinobacteria | 0.24%±0.30% | 0.32%±0.45% | t=-0.35 | p=0.74 | 0.69%±1.44% | 0.23%±0.08% | t=0.72 | p=0.51 |
| Cyanobacteria | 0.21%±0.15% | 0.39%±0.37% | t=-1.02 | p=0.35 | 0.48%±0.23% | 0.34%±0.22% | t=0.93 | p=0.38 |
| Spirochaetes | 0.13%±0.12% | 0.16%±0.24% | t=-0.21 | p=0.84 | 0.25%±0.24% | 0.38%±0.59% | t=-0.45 | p=0.66 |
| Lentisphaerae | 0.11%±0.11% | 0.14%±0.15% | t=-0.31 | p=0.77 | 0.51%±0.54% | 0.14%±0.16% | t=1.49 | p=0.20 |
| Fusobacteria | 0.005%±0.01% | 0.03%±0.05% | t=-1.26 | p=0.27 | 0.03%±0.05% | 0.44%±0.98% | t=-0.92 | p=0.41 |

Table S5 The differences in relative abundance (mean ± SD) of major bacterial phyla between male and female forest musk deer

| phylum level | Juvenile | |  | | Adult | |  | |
| --- | --- | --- | --- | --- | --- | --- | --- | --- |
|  | MF | FF |  |  | MF | FF |  |  |
| Firmicute | 71.15%±16.00% | 55.90%±16.24% | t=1.50 | p=0.17 | 55.66%±20.53% | 58.66%±9.36% | t=-0.30 | p=0.78 |
| Bacteroidetes | 17.02%±8.71% | 19.17%±6.34% | t=-0.45 | p=0.67 | 12.59%±4.07% | 20.65%±7.31% | t=-2.15 | p=0.06 |
| Proteobacteria | 10.35%±16.63% | 19.43%±20.08% | t=-0.78 | p=0.46 | 28.67%±23.97% | 17.45%±13.37% | t=0.91 | p=0.40 |
| Verrucomicrobia | 0.17%±0.17% | 2.57%±3.91% | t=-1.37 | p=0.24 | 0.78%±1.02% | 0.61%±0.56% | t=0.34 | p=0.74 |
| Tenericutes | 0.58%±0.30% | 0.90%±0.56% | t=-1.10 | p=0.30 | 1.17%±0.76% | 1.01%±1.06% | t=0.27 | p=0.80 |
| Actinobacteria | 0.24%±0.30% | 0.69%±1.44% | t=-0.69 | p=0.53 | 0.32%±0.45% | 0.23%±0.08% | t=0.48 | p=0.65 |
| Cyanobacteria | 0.21%±0.15% | 0.48%±0.23% | t=-2.17 | p=0.06 | 0.39%±0.37% | 0.34%±0.22% | t=0.26 | p=0.80 |
| Spirochaetes | 0.13%±0.12% | 0.25%±0.24% | t=-0.99 | p=0.35 | 0.16%±0.24% | 0.38%±0.59% | t=-0.78 | p=0.46 |
| Lentisphaerae | 0.11%±0.11% | 0.51%±0.54% | t=-1.64 | p=0.17 | 0.14%±0.15% | 0.14%±0.16% | t=-0.40 | p=0.97 |
| Fusobacteria | 0.005%±0.01% | 0.03%±0.05% | t=-1.28 | p=0.26 | 0.03%±0.05% | 0.44%±0.98% | t=-0.92 | p=0.41 |

Table S6 The differences in relative abundance (mean ± SD) of major bacterial genera between Juvenile and Adult forest musk deer

| genus level | MF | |  | | FF | |  | |
| --- | --- | --- | --- | --- | --- | --- | --- | --- |
|  | Juvenile | Adult |  |  | Juvenile | Adult |  |  |
| *Ruminococcaceae | 26.97%±11.02% | 20.01%±7.48% | t=1.17 | p=0.28 | 21.75%±7.10% | 22.70%±6.06% | t=-0.23 | p=0.83 |
| *Clostridiales | 13.27%±0.46% | 10.48%±5.04% | t=0.91 | p=0.39 | **13.75%±5.30%** | 11.69%±5.08% | t=0.63 | p=0.55 |
| Pseudomonas | 7.43%±16.60% | 12.90%±18.69% | t=-0.49 | p=0.64 | 2.48%±5.41% | 12.31%±11.69% | t=-1.71 | p=0.14 |
| *Lachnospiraceae | 7.18%±4.00% | 7.77%±5.94% | t=-0.18 | p=0.86 | 6.21%±4.37% | 8.02%±1.73% | t=-0.86 | p=0.43 |
| Dorea | 9.21%±7.37% | 6.13%±3.05% | t=0.87 | p=0.41 | 3.26%±2.49% | 7.02%±4.77% | t=-1.56 | p=0.16 |
| *Bacteroidales | 5.18%±3.87% | 3.10%±2.61% | t=1.00 | p=0.35 | 9.42%±8.78% | 6.34%±3.86% | t=0.72 | p=0.50 |
| *Enterobacteriaceae | 1.91%±4.13% | 8.94%±11.54% | t=-1.28 | p=0.26 | 9.12%±12.46% | 0.11%±0.11% | t=1.62 | p=0.18 |
| 5-7N15 | 7.80%±4.79% | 3.49%±1.87% | t=1.88 | p=0.10 | 3.34%±1.56% | 4.21%±2.78% | t=-0.61 | p=0.56 |

Table S7 The differences in relative abundance (mean ± SD) of major bacterial genera between male and female forest musk deer

| genus level | Juvenile | |  | | Adult | |  | | |
| --- | --- | --- | --- | --- | --- | --- | --- | --- | --- |
|  | MF | FF |  |  | MF | FF |  |  |  |
| *Ruminococcaceae | 26.97%±11.02% | 21.75%±7.10% | t=0.89 | p=0.40 | 20.01%±7.48% | 22.70%±6.06% | t=-0.63 | p=0.55 |  |
| *Clostridiales | 13.27%±4.64% | 13.75%±5.30% | t=-0.15 | p=0.88 | 10.48%±5.05% | 11.69%±5.08% | t=-0.38 | p=0.71 | |
| Pseudomonas | 7.43%±16.60% | 2.48%±5.41% | t=0.63 | p=0.54 | 12.90%±18.69% | 12.31%±11.69% | t=0.06 | p=0.95 | |
| *Lachnospiraceae | 7.18%±4.00% | 6.21%±4.37% | t=0.37 | p=0.72 | 7.77%±5.94% | 8.02%±1.73% | t=-0.09 | p=0.93 | |
| Dorea | 9.21%±7.37% | 3.26%±2.49% | t=1.71 | p=0.12 | 6.13%±3.05% | 7.02%±4.77% | t=-0.35 | p=0.74 | |
| *Bacteroidales | 5.18%±3.87% | 9.42%±8.78% | t=-0.99 | p=0.37 | 3.10%±2.61% | 6.34%±3.86% | t=-1.56 | p=0.16 | |
| *Enterobacteriaceae | 1.91%±4.13% | 9.12%±12.46% | t=-1.23 | p=0.25 | 8.94%±11.54% | 0.11%±0.11% | t=1.71 | p=0.16 | |
| 5-7N15 | 7.80%±4.79% | 3.34%±1.56% | t=1.98 | p=0.08 | 3.49%±1.87% | 4.21%±2.78% | t=-0.48 | p=0.64 | |


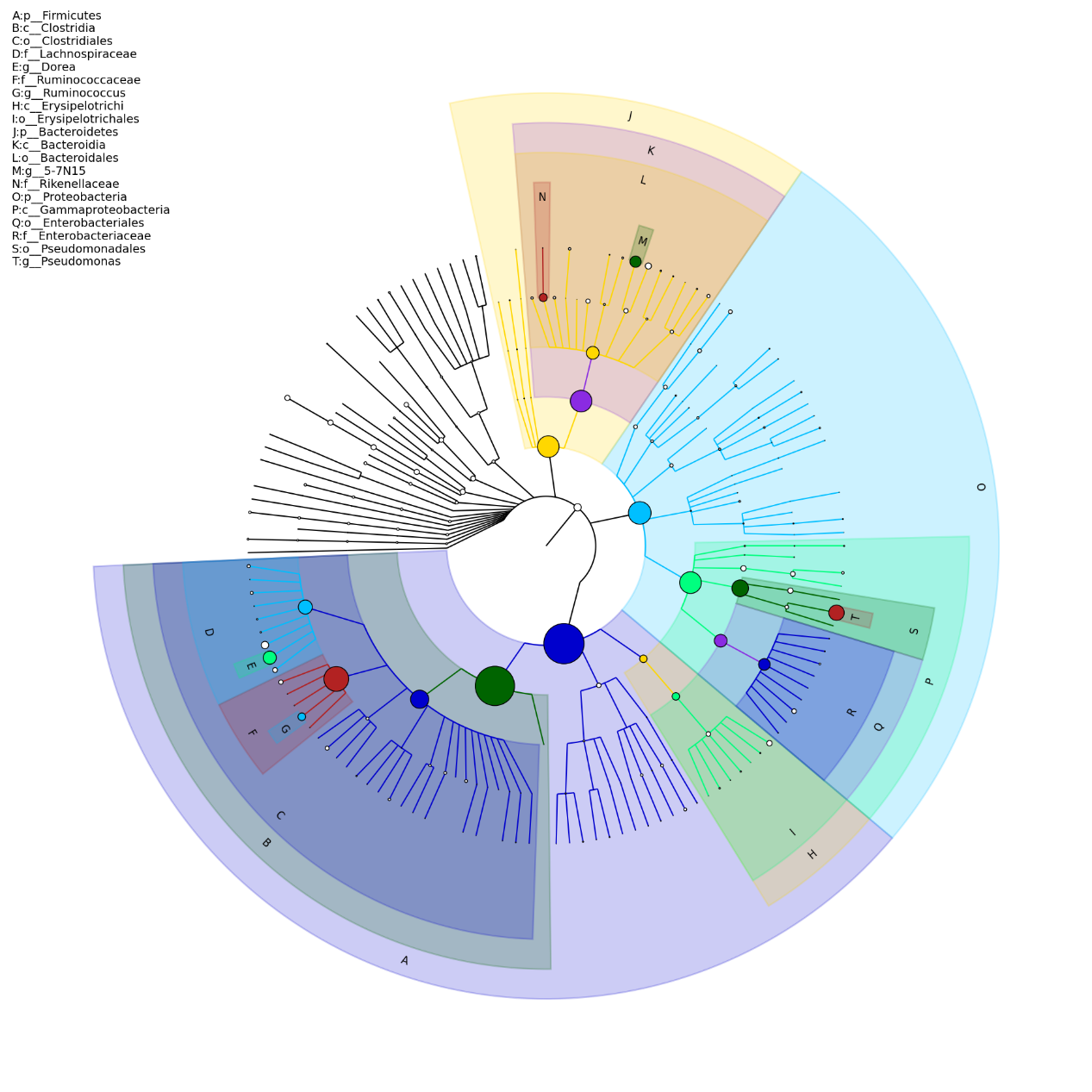


Figure S1 Classification tree by GraPhlAn

The node size corresponds to the average relative abundance of the taxa and the classification of the relative abundance of the top 20 species
